# Association of necrotizing enterocolitis with antimicrobial exposure in preterm infants <32 weeks gestational age:A multicenter prospective case-control study

**DOI:** 10.3389/fphar.2022.976487

**Published:** 2022-09-23

**Authors:** Xiaojing Pei, Yujun Gao, Yan Kou, Yanjie Ding, Dan Li, Peng Lei, Lili Zuo, Qiongyu Liu, Naiying Miao, Simmy Reddy, Yonghui Yu, Xuemei Sun

**Affiliations:** ^1^ Department of Neonatology, Linyi People’s Hospital, Linyi, China; ^2^ Department of Neonatology, Baogang Third Hospital of Hongci Group, Baotou, China; ^3^ Department of Neonatology, The First Affiliated Hospital of Shandong First Medical University, Jinan, China; ^4^ Department of Neonatology, Yantai Yuhuangding Hospital, Yantai, China; ^5^ Department of Neonatology, Liaocheng People’s Hospital, Liaocheng, China; ^6^ Department of Neonatology, Jinan Maternity and Child Care Hospital, Jinan, China; ^7^ Department of Neonatology, Zibo Maternity and Child Care Hospital, Zibo, China; ^8^ Department of Neonatology, Women and Children’s Healthcare Hospital of Linyi, Linyi, China; ^9^ Department of Neonatology, Hebei Petro China Central Hospital, Hebei, China; ^10^ Cheeloo College of Medicine, Shandong University, Jinan, China; ^11^ Department of Neonatology, Shandong Provincial Hospital, Shandong University, Jinan, China; ^12^ Department of Neonatology, Shandong Provincial Hospital affiliated to Shandong First Medical University, Jinan, China

**Keywords:** necrotizing enterocolitis, very preterm infants, multicenter, antimicrobial exposure, case-control study

## Abstract

**Objective:** To assess the risk of necrotizing enterocolitis (NEC) and explore the relationship between antibiotic overexposure and disease occurrence in a large prospective birth cohort.

**Methods:** Based on a prospective birth cohort, the study collected hospitalization data of very preterm infants (VPIs) having gestational age of less than 32 weeks from January 1, 2018, to June 30, 2021 via the China Northern Neonatal Network. Infants diagnosed with NEC ≥ stage II were included in the case group, and each case was matched for GA and birth weight for the control group. Furthermore, the risk factors for NEC were determined by statistical analyses.

**Results:** A total of 6425 VPIs were included in this study, and 167 (2.6%) of these subjects were diagnosed with NEC ≥ stage II. The study also included 984 extremely preterm infants (gestational age <28 weeks), including 50 (5.1%) infants diagnosed with NEC ≥ stage II. In the matched case-control study, subjects had a total of antibiotic days-of-therapy for 9015 days, of which broad-spectrum antibiotics (BSAs) accounted for 77%. The antibiotic spectrum index per antibiotic day in the case group was significantly higher and was an independent risk factor for the occurrence of NEC (*p* = 0.001, *OR* = 1.13).

**Conclusion:** The cohort of VPIs was overexposed to antiboitics. Unreasonable combination of antibiotics and overexposure to BSAs may increase the risk of NEC in preterm infants.

## Introduction

Necrotizing enterocolitis (NEC) is a severe form of gastrointestinal infection that affects preterm babies in the majority of cases, and very preterm infants (VPIs) are at the highest risk of developing NEC. The incidence rate of NEC is about 2–6% (Battersby et al., 2018), while the morbidity and mortality rates for surgical NEC are reported as high as 30–50% in the neonatal intensive care units (NICUs) (Kiechl-Kohlendorfer et al., 2019; Flahive et al., 2020). Following its onset, NEC progresses insidiously in the initial phase, but an aggressive disease progression can be observed in the later phases. The clinical manifestations of NEC are often systemic such as abdominal distension, vomiting, bloody stool, and septic shock. The pathogenesis of NEC involves multifactorial risk factors, including autoimmunity, enteral feeding, intestinal microbial imbalance, ischemic hypoxic injury, and microbial infections. The small gestational age and low birth weight have been recognized as the major risk factors for NEC (Samuels et al., 2017; Baranowski and Claud, 2019).

The healthy gut microbiome is central to neonatal mucosal immunity and proper gut development. Notably, the use of antibiotics in the early postnatal period overlaps with the critical window of neonatal gut microbial colonization. Undoubtedly, long-term application and/or antibiotic overdosing can significantly hinder the normal physiological process of gut microbiome development in neonates due to the antibiotic-mediated killing of beneficial bacteria like Bifidobacteria in the intestine (Roze et al., 2017). An unbalanced gut microbiome further contributes to potentially fatal preterm birth diseases, such as NEC (Lange et al., 2016), under the influence of other risk factors.

The Chinese Adverse Prognosis of VPIs (CARE-Preterm) cohort study involves the multicenter prospective dynamic observational cohorts of preterm infants, with data from a Sino-northern Neonatal Network (SNN) of 46 participating NICUs across six provinces and autonomous regions of northern China having a population of 120 million. Moreover, a large research-based database was dedicated to collecting real-world data and optimizing clinical management. According to our previous published studies (Dong, 2020), NEC is considered the leading cause of death of extremely preterm infants (GA ＜28 weeks) in the middle and late stages of hospitalization. The majority of preterm infants in this cohort reported having excessively prolonged antibiotic use, even with the combination of third-generation (e.g., cephalosporins) and broad spectrum (e.g., carbapenems) in some instances (Hou et al., 2022). Therefore, the present study was designed based on this perspective preterm birth cohort to investigate the relationship between pre-onset antibiotic use and morbidity in the NEC population of VPIs.

## Materials and methods

### Study design and subjects

This study was a multicenter, prospective, dynamically observed case-control study of the CARE-Preterm cohort of VPIs in China. The live births having GA of <32 weeks who were admitted to the participating units from January 1, 2018, to June 30, 2021 were selected for this investigation. Required data were retrieved from the SNN database.

### Criteria and grouping

The study inclusion criteria were as follows: 1) GA at birth should be <32 weeks; and 2) the preterm neonate was admitted to the hospital within 24 h of live birth. The exclusion criteria included: 1) diagnosis with congenital gastrointestinal tract malformation; 2) congenital or inherited metabolic disorders; and 3) incomplete patient data.

Children diagnosed with NEC ≥ stage II by Bell staging during the hospitalization were included in the case group, and each child was matched with 3 non-NEC infants according to their GAs (±3 days) and birth weights (±100 g), which constituted the control group.

### Definitions

Length-of-therapy (LOT) was defined as the number of days a patient received antibiotics, irrespective of the variety of antibiotics. Days-of-therapy (DOT) was defined as the accumulated number of days of systemic antibiotic therapy (Flannery and Horbar, 2020). Both LOT and DOT were described in relation to each research object in this study.Antibiotic spectrum index (ASI) was defined as a novel metric accounting for the spectrum of activities of a drug or antibiotic (Gerber et al., 2017). ASI assigned points for activities of an antibiotic or combination of antibiotics against the clinically relevant pathogens, ranging from 1 to 13 for each drug, with a higher ASI being reflective of a more broad spectrum agent. For example, an infant treated with penicillin combined with cefotaxime for 3 days had an ASI value of 2 for penicillin and a value of 5 for cefotaxime. Hence, the LOT of the child was 3 days, the DOT was 6 days, and the mean ASI per antibiotic day was 7 (21/3).Antibiotic use rate (AUR) is defined as the number of days an infant was exposed to 1 or more antibiotics divided by the age on the day before onset (Gerber et al., 2017). Broad-spectrum antibiotics (BSAs) include third-generation cephalosporins, carbapenems and part of the penicillin like piperacillin-tazobactam.

Small for gestational age (SGA) was defined as a birth weight of less than 10th percentile for gestational age. Amniotic fluid pollution refers to the entry of meconium and other components into the amniotic fluid, causing the amniotic fluid to be turbid and abnormal in composition. Early onset sepsis (EOS) defined by isolation of pathogenic species from blood or cerebrospinal fluid culture within 72 h of birth. Bronchopulmonary dysplasia (BPD) was defined as oxygen requirement at 36 weeks of post-menstrual age (Ehrenkranz et al., 2005). Hemodynamically significant patent ductus arteriosus (hsPDA) was defined as an echocardiographic confirmed PDA for which pharmacological (ibuprofen, indomethacin) or surgical treatment was initiated. All VPIs with hsPDA in our cohort received drug treatment first. After that, the decision to accept surgery is based on the effect of drug treatment and the physical fitness of the child. Extrauterine growth restriction (EUGR) was defined as having a measured growth value (weight, length or head circumference) that was <10th percentile of the predicted value when the premature neonates discharged from the hospital (Clark et al., 2003).Parenteral nutrition-associated cholestasis (PNAC) is defined as a direct bilirubin of 34.2 μmol/L or greater while being on parenteral nutrition for 14 or more days without other potential causes (Satrom and Gourley, 2016).

### Variable collection

The clinical data of each subject during hospitalization was collected that included- 1) patient demographics such as birth weight, GA, gender, SGA, multiple birth, postnatal Apgar score, admission temperature; 2) perinatal factors like prenatal hormone application, maternal hypertensive disorders of pregnancy (HDP), amniotic fluid contamination, duration of premature rupture of membranes (PROM) ≥18h, antibiotics used within 24 h of delivery; 3) NICU treatment records including days of invasive and non-invasive mechanical ventilation (the modes mainly include NIPPV and CPAP), use of pulmonary surfactant, age for starting intestinal feed, type of dairy feeding, and antibiotic use strategy; and (4) Complications during hospitalization: respiratory distress syndrome (RDS), EOS, BPD, EUGR and PNAC.

NICU administration data were collected to the day before the onset of NEC in the case group.The data of the control group were collected to the same postnatal days.

### Statistical methods

All data was statistically analyzed using SPSS 26.0 software. Non-normal measures were expressed as medians and quartiles, and comparisons between groups were made using the Wilcoxon test or Kruskal–Wallis test; count data sets were expressed as cases (%), and comparisons between groups were made using the chi-square test or Fisher’s exact probability method. Variables that were not used as matching factors were subjected to one-way analysis, and those with statistically significant results were included in the multi-factor logistic analysis. Conditional logistic regression analysis was performed on the matched data to analyze the risk factors for the occurrence of NEC, and *p* < 0.05 was considered a statistically significant difference.

## Results

**FIGURE 1:**
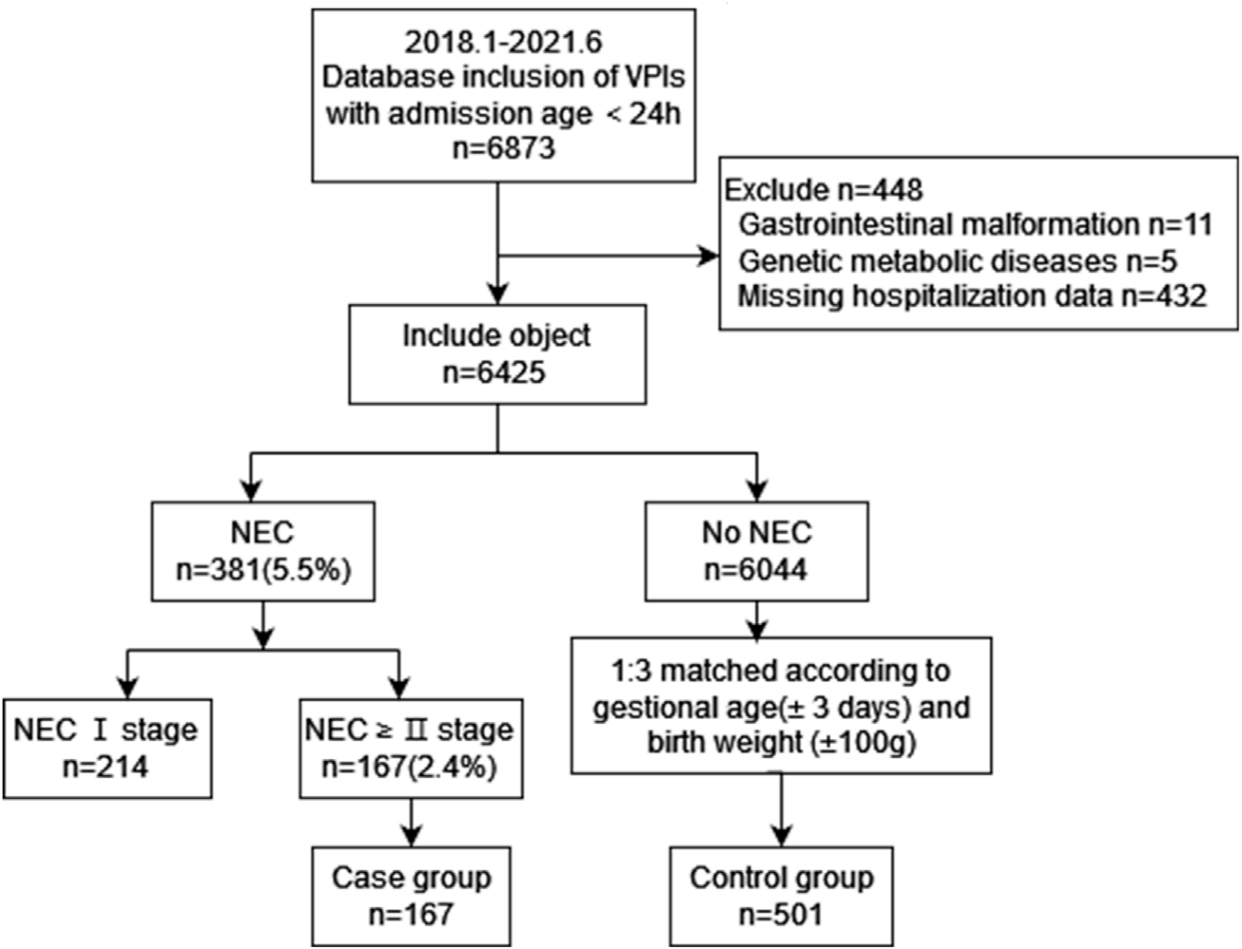
Inclusion flow chart.

A total of 6425 VPIs were included in this study, and 167 (2.6%) neonates were diagnosed with NEC ≥ stage II. In this cohort, 984 extremely preterm infants (EPIs) were included, of which 50 (5.1%) neonates were diagnosed with NEC ≥ stage II. The mean age of onset of NEC was 19±12 days after birth. The inclusion process is shown in Figure 1.

Demographic and perinatal information for infants with NEC ≥ stage II or non-NEC revealed significant differences between the two groups in terms of GA and birth weight (both *p* <0.001) before matching. Based on the GA and birth weight, the control group was formed with 501 non-NEC infants. The two groups were well matched for GA and birth weight. The admission temperature was significantly lower in the case group as compared to that of the control group (*p* = 0.04) (Table 1).

**TABLE 1:** Comparison of general data and perinatal factors before and after matching.

Variables	Total		*P*	Matched infants		*p*
	Non-NEC (N = 6044)	NEC ≥ II stage (N = 167)		Controls (n = 501)	Cases (n = 167)	
GA (weeks), median [IQR]	30 (29;31)	29 (27;30)	<0.001	29 (27;30)	29 (27;30)	0.75
Birth weight(g), median [IQR]	1300 (1100;1550)	1190 (925;1400)	<0.001	1170 (950;1400)	1190 (925;1400)	0.82
Male, n (%)	3325 (55.0)	98 (58.7)	0.35	270 (53.9)	98 (58.7)	0.28
SGA, n (%)	242 (4.0)	5 (3.0)	0.51	12 (2.4)	5 (3.0)	0.78
Admission temperature (°C), median [IQR]	36 (36;36)	36 (36;36)	0.50	36 (36;36)	36 (36;36)	0.04
Multiple births, n (%)	1392 (23.0)	44 (26.4)	0.32	136 (27.2)	44 (26.4)	0.84
Assisted Reproduction, n (%)	737 (12.2)	29 (17.4)	0.05	75 (15.0)	29 (17.4)	0.46
Cesarean, n (%)	3951 (65.4)	102 (61.1)	0.25	329 (65.7)	102 (61.1)	0.28
1min Apgar score, median [IQR]	8 (6;9)	7 (5;9)	0.03	8 (6;9)	7 (5;9)	0.06
5min Apgar score, median [IQR]	8 (7;9)	8 (7;9)	0.04	8 (7;9)	8 (7;9)	0.08
Contaminated amniotic fluid, n (%)	332 (5.5)	8 (4.8)	0.69	18 (3.6)	8 (4.8)	0.49
PROM≥18h, n (%)	1304 (21.6)	47 (28.1)	0.04	133 (26.6)	47 (28.1)	0.69
HDP, n (%)	1561 (25.8)	39 (23.4)	0.47	141 (28.1)	39 (23.4)	0.23
Antenatal hormone use, n (%)	4230 (70.0)	138 (82.6)	<0.001	393 (78.4)	138 (82.6)	0.25
Prenatal antibiotic use, n (%)	2167 (35.9)	66 (39.5)	0.33	202 (40.3)	66 (39.5)	0.86

GA, gestational age; SGA, small for gestational age; PROM, premature rupture of membranes; HDP, hypertensive disorders of pregnancy.

An in-depth analysis of the antibiotic use between these two groups showed that the total LOT of the two groups was 7177 days, accounting for 59% of the total observation period, and the LOT per infant was 11±8 days. The total DOT of the included populations was 9015 days, and the DOT per infant was 14±11 days. The DOT of BSAs was 6920 days, accounting for 77% of the total DOT. The case group had higher ASI per antibiotic day (*p* = 0.006), which measured the breadth of the antimicrobial spectrum. However, there were no significant differences in LOT, DOT, and AUR between the two groups (Table 2). Additionally, the duration of mechanical ventilation in the case group was relatively longer than that of the control group (*p* = 0.03). Enteral feeding was initiated earlier in the control group (*p* = 0.006).

**TABLE 2:** Description and single factor analysis of NICU management in matched populations.

Variables	Controls (n = 501)	Cases (n = 167)	*p*
LOT (days), median [IQR]	10 (6;15)	10 (6;15)	0.94
DOT (days), median [IQR]	11 (7;20)	13 (7;20)	0.14
AUR (%)	79.0 (46.7;100.0)	68.5 (44.7;100.0)	0.37
ASI per antibiotic days [IQR]	8 (6;9)	8 (7;10)	0.006
BSAs DOT (days), median [IQR]	10 (6;14)	10 (6;15)	0.65
EOS, n (%)	103 (20.6)	34 (20.4)	0.96
Invasive mechanical ventilation (days), median [IQR]	0 (0;5)	3 (0;5)	0.03
Non-invasive ventilation (days), median [IQR]	8 (4;15)	9 (4;17)	0.36
Pulmonary surfactant, n (%)	344 (68.6)	124 (74.2)	0.17
Age for starting intestinal feed (days), median [IQR]	1 (0;2)	1 (1;2)	0.006
*Early postnatal formula feeding, n (%)	399 (79.6)	141 (84.4)	0.17
Fasting days (days), median [IQR]	0 (0;0)	0 (0;1)	0.36
Red-blood cell transfusions, n (%)	181 (36.1)	63 (37.7)	0.71

LOT, length of therapy; DOT, days of therapy; AUR, antibiotic use rate; BSAs, broad spectrum antibiotics; ASI, antibiotic spectrum index; EOS, early onset sepsis.*Subjects were either formula-fed or breastfed their own mother.

In the analysis of the types of antibiotics used, the third-generation cephalosporin was the most frequently prescribed medication (36.5% vs. 30.1%), but there was no significant difference between the two groups (*p* = 0.45). The number of total prescriptions in the case group was significantly higher than that in the control group (*p* = 0.04), as shown in Table 3.

**TABLE 3:** The usage of BSAs usage in case and control group.

Drug class	Duration of prescribed antibiotics, n (%)		*p*	Days of therapy (days/infant),median [IQR]		*p*
	Controls	Cases		Controls	Cases	
Third-generation cephalosporins	314 (36.5)	96 (30.1)	0.45	0 (0;3)	0 (0;7)	0.65
Carbapenems	144 (16.7)	57 (17.9)	0.17	0 (0;0.3)	0 (0;0)	0.29
Broad spectrum penicillin	219 (25.4)	77 (24.1)	0.59	6 (4;9)	7 (4;11)	0.06
Total prescriptions	861 (100)	319 (100)	0.04	10 (5;16)	11 (6;18)	0.11

Inclusion of statistically different variables between the two groups after pairwise univariate analysis in the conditional logistic regression model revealed that higher ASI per antibiotic d was an independent risk factor for the development of NEC, with each 1-unit increase in ASI per antibiotic days associated with a 0.13-fold increase in the risk of developing NEC (Table 4).

**TABLE 4:** Conditional Logistics regression analysis of NEC risk factors.

Variables	*p* value	*OR*	95%CI	
ASI per antibiotic days	0.001	1.13	1.05	1.22
Admission temperature	0.18	0.82	0.61	1.10
Invasive mechanical ventilation	0.65	1.01	0.97	1.06
Age for starting intestinal feed	0.07	1.05	1.00	1.11

Comorbidity and death of preterm infants were higher in the case group as compared to those of the control group. Moreover, the extrauterine growth restriction (EUGR), parenteral nutrition-associated cholestasis (PNAC), and death were significantly associated with the occurrence of NEC in the multifactorial logistic regression analysis (*p* < 0.05) in Table 5.

**TABLE 5:** Studies on the correlation between NEC and hospitalization outcomes.

	Controls (n = 501) n (%)	Cases (n = 167) n (%)	*P* ^*a*^	*P* ^*b*^	*Ajusted* ^*^ *OR*
RDS	418 (83.4)	143 (85.6)	0.50	0.82	1.03
hsPDA	96 (19.1)	23 (13.7)	0.12	0.08	0.60
Moderate-severe BPD	61 (13.2)	32 (19.2)	0.02	0.80	1.01
EUGR	129 (25.8)	64 (38.3)	0.002	0.03	1.62
PNAC	58 (11.6)	55 (32.9)	<0.001	<0.001	4.20
Death	13 (2.6)	41 (24.6)	<0.001	<0.001	16.25

RDS, respiratory distress syndrome; hsPDA, hemodynamically significant patent ductus arteriosus; BPD, bronchopulmonary dysplasia; EUGR, extrauterine growth restriction; PNAC, parenteral nutrition-associated cholestasis.

^a^ is single-factor analysis;^b^ is multi-factor logistic analysis;^*^ The correction factors were ASI, per antibiotic days.

## Discussion

The study was based on a large prospective preterm birth cohort in northern China with real-world data collection. In this study, the rate of incidence of NEC at stage II or above in VPIs was 2.6%, while that in EPIs was 5.1%. These incidence rates were similar to that of Austria (Kiechl-Kohlendorfer et al., 2019), whose rates of local incidences of severe NEC among VPIs and EPIs were 2.1% and 4.4%, respectively. However, the French EPIPAGE-2 cohort study showed a 3.7% incidence rate for NEC with ≥stage II in surviving preterm infants at 23–31 weeks of GA (Roze et al., 2017). The rate of incidence was also lower in the Japanese regions, with a prevalence of 1.6% for severe NEC cases (Isayama et al., 2012). The differences in disease incidence rates might be related to differences in the structure of the included patient populations, level of care, and diagnostic criteria.

We delved into the details of antibiotic use between the case and control groups which indicated that the LOT of antibiotic use in both groups was 10 (IQR: 6;15) days. During an average observation period of 19 days, the rate of exposure to antibiotics was almost 60%. Antibiotic exposure was generally long, with no significant differences between the two groups. The DOT was 13 (IQR: 7;20) days for the case group and 11(IQR: 7;20) days for the control group. The DOT was longer than the LOT in both groups, suggesting a common antibiotic combination strategy in this cohort. These results were consistent with the findings of the previous investigations (Hou et al., 2022) conducted by the same collaborative group. Seventy-five percent of hospitalized very low birth weight infants (birth weight <1500 g) received post-birth antibiotic treatments. Among them, 78.0% had a course of more than 7 days, and the use of 3 or more antibiotics increased significantly in extended courses. Compared with reports from France and the United States (Flannery et al., 2018; Martin-Mons et al., 2020), prolonged antibiotic exposure is a clinical phenomenon that cannot be ignored in this cohort. According to the analysis of the type of antibiotics used, more than 70% of DOTs were caused by broad-spectrum antibiotics. The most frequently prescribed antibiotic in these NICUs was the third generation cephalosporins, followed by carbapenems which contributed to 17% of all antibiotic prescriptions, which was significantly higher than in India (4.5%) (Gandra et al., 2017) and Australia (3.2%) (Osowicki et al., 2014).

ASI is a novel antibacterial metric that measures the breadth of antibiotic exposure. This study is the first to introduce the ASI to observe the association of broad-spectrum antibiotics with the occurrence of NEC. Compared with preterm infants without NEC in the control group, the ASI per antibiotic day was higher in the case group and was independently related to the occurrence of the disease, indicating that children with NEC may have a higher exposure to broader bactericidal spectrum prior to the disease onset. On the one hand, patients treated with BSAs in the early postnatal period may have severe disease symptoms and a higher risk of NEC. After the pathogen invades the intestinal tract of VPIs, it produces endotoxins like bacterial lipopolysaccharide (LPS) and lipoteichoic acid (LTA) that cause direct damage to the intestinal tract. It also causes a cascade of inflammatory responses by producing a variety of cellular pro-inflammatory factors, resulting in persistent damage to the intestinal wall (De Plaen, 2013; Molteni et al., 2016). However, our previous study found that the rate of incidence of EOS in infants younger than 32 weeks of GA was 1.8%, which was not significantly higher than that in high-income countries (Dong, 2020). The effect of a high rate of BSAs exposure and prolonged antibiotic usage cannot be fully explained by the higher infection rate. The prescriber’s subjective decision may be the main cause of this clinical phenomenon.

On the other hand, the overexposure to antibiotis reflected by the high ASI per antibiotic days could itself be a risk factor for an increased rate of disease occurrence. Intestinal dysbiosis is considered the key link in the pathogenesis of NEC. The expansion of the antimicrobial spectrum exacerbates the reduction in the biodiversity of the intestinal microbiota, while the ability of the intestinal barrier to prevent the invasion of exogenous and potentially pathogenic microorganisms is also potentially suppressed (Sun et al., 2021). Several studies have found that the prolonged use of empirical antibiotics was associated with an increase in the incidence of NEC (Alexander et al., 2011; Kuppala et al., 2011). In addition, infants treated with broad-spectrum antibiotics amoxicillin and gentamicin had a significantly higher incidence of NEC (Raba et al., 2021). In this cohort, combination of antibiotics and broad-spectrum antibiotics might have led to an expansion of the antimicrobial spectrum compared to the usually longer duration of antibiotic use. It is reflected by ASI and is significantly associated with the onset of NEC. Additionally, the expansion of the antimicrobia spectrum might shift antimicrobial resistance gene profile directly following treatment (Reyman et al., 2022). The over-use of third-generation cephalosporins and carbapenems is also an ongoing global public threat. In a previous study (Hou et al., 2022), we have shown that approximately half of the Gram-negative pathogens in hospitalized preterm infants with EOS are resistant to third-generation cephalosporins. This dire clinical reality reflects the severity of antibiotic over-use in neonates. Clinicians are advised not only to limit the number of days of antibiotic use, but also to reduce unreasonable antibiotic combinations and chronic exposure to BSAs, thereby limiting the incidence of other comorbidities, such as NEC in VPIs.

NEC is a multifactorial disease. This study only matched gestational age and birth weight, which had a significant impact on disease occurrence. Since this study could not cover all other aspects of NEC pathogenesis in preterm infants, it inevitably included selection bias. However, the study was real-world study based on a prospective birth cohort with data from a clinical research database. The ASI was introduced to delineate the possible causal relationship between severe antibiotic overexposure and NEC onset in VPIs. At the same time, the clinical data have been collected from multiple centers across China, which can truly reflect differences in the use of antibiotics between the low-and middle-income countries.

## Conclusion

Our study describes exposure to the antimicrobial spectrum and duration of antibiotics before onset in preterm infants with NEC, and highlights the severity of antibiotic over-use. Unreasonable combination of antibiotics and overexposure to broad-spectrum antibiotics may increase the risk of NEC in preterm infants. Moreover, this study provides with the baseline data for improving the quality of antibiotic treatment, optimizing the clinical management of premature infants.

## Data Availability

The raw data supporting the conclusion of this article will be made available by the authors, without undue reservation.
